# A Lecithin Liposome Stimulates Soil Microbial Respiration and Nitrate Immobilization

**DOI:** 10.1021/acsagscitech.5c00587

**Published:** 2025-11-24

**Authors:** Camille R. Butkus, Julie N. Weitzman, Alireza Mohammadzadeh, Patrick J. Dunn, Jason P. Kaye, Leanne M. Gilbertson, Steven R. Little, Emily M. Elliott

**Affiliations:** † Department of Geology and Environmental Science, 6614 University of Pittsburgh, Pittsburgh, Pennsylvania 15260, United States; ‡ Department of Bioengineering, 6614 University of Pittsburgh, Pittsburgh, Pennsylvania 15260, United States; § Department of Civil and Environmental Engineering, 6614 University of Pittsburgh, Pittsburgh, Pennsylvania 15260, United States; ∥ Department of Ecosystem Science and Management, The Pennsylvania State University, University Park, Pennsylvania 16802, United States; ⊥ Department of Civil and Environmental Engineering, 3065 Duke University, Durham, North Carolina 27708, United States; # Department of Department of Chemical and Petroleum Engineering, 6614 University of Pittsburgh, Pittsburgh, Pennsylvania 15260, United States; ∇ Department of Clinical and Translational Science, 6614 University of Pittsburgh, Pittsburgh, Pennsylvania 15260, United States; ○ McGowan Institute for Regenerative Medicine, 6614 University of Pittsburgh, Pittsburgh, Pennsylvania 15260, United States; ◆ Department of Immunology, 6614 University of Pittsburgh, Pittsburgh, Pennsylvania 15260, United States; ¶ Department of Pharmaceutical Sciences, 6614 University of Pittsburgh, Pittsburgh, Pennsylvania 15260, United States; & Department of Ophthalmology, 6614 University of Pittsburgh, Pittsburgh, Pennsylvania 15260, United States

**Keywords:** microbial nitrogen immobilization, lipid vesicle, soil microbes, nitrogen cycling, agriculture

## Abstract

Liposomes are microscale lipid vesicles used in pharmaceuticals, food products, and most recently, agriculture. Several studies have shown that liposomes can deliver nutrients to plant leaves, often more efficiently than traditional forms. However, the delivery of plant nutrients to soil via liposomes remains understudied. Interactions between liposomes and soil microbes, including metabolism of the lipid carbon (C) and assimilation of liposome-encapsulated nutrients into soil microbial biomass, could alter the availability of nutrients within the soil. We assessed the impact of lecithin liposomes with nitrogen (N) cargo on C and N cycling during a 7-day incubation experiment. We quantified changes in concentrations of carbon dioxide, nitrous oxide, oxygen, and soil inorganic N pools including soil extractable nitrate (NO_3_^–^-N) and ammonium (NH_4_^+^-N). Liposome additions increased microbial respiration and resulted in rapid soil NO_3_^–^-N immobilization, suggesting that liposomes may be a tool to immobilize N and reduce agricultural N losses.

## Graphical abstract

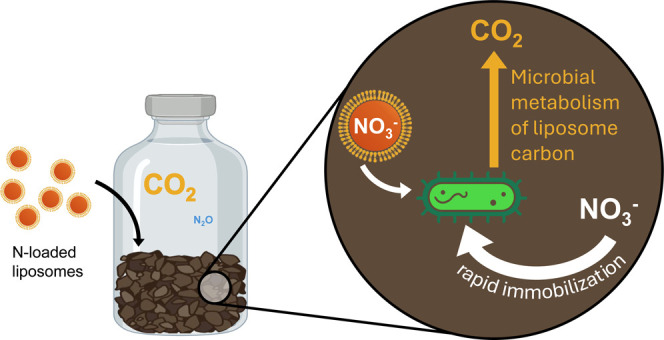

## Introduction

### Liposomes as Potential Agrochemical Carriers

Liposomes are well-established as carriers of pharmaceutical drugs and functional food additives, serving as a protected cargo-delivery system that can transport encapsulated compounds to targeted sites, often enabling controlled or sustained release.^1,2^ These manufactured lipid vesicles range from 0.025 to 2.5 μm in diameter and consist of single or double phospholipid membranes, making them both biodegradable and biocompatible.^3^ Phospholipids consist of a phosphate-containing polar, hydrophilic head attached to two nonpolar, hydrophobic fatty acid chains by a three-carbon glycerol backbone. Liposomes can be single or multilayered, with concentric bilayers or smaller liposomes encapsulated within larger liposomes. Their versatility lies in their ability to compartmentalize hydrophobic cargo within the lipid layer and hydrophilic cargo in the aqueous core.^4^ Additionally, liposome properties, including charge, size, permeability, stability, and responsiveness to pH or temperature, are tunable through lipid composition and production method.^5–9^ These properties position liposomes as a highly adaptable platform for exploring cargo delivery across diverse systems.

Building on this versatility, liposomes may also be well-suited for delivering agrochemical loads to soils and crops, with the potential to enhance the efficiency of agrochemical delivery.^5,7,10–16^ They have already been used as dispersal agents for pesticides, carriers for both herbicides and pesticides applied to crops, and carriers to deliver nutrients to plant leaves.^17–22^ In one study, liposomes bound to montmorillonite (i.e., clay) particles significantly reduced the leaching of their cargo, sulfometuron and sulfosulfuron, in soil columns.^22^ When applied as a foliar spray, up to 33% of iron (Fe) and magnesium (Mg) encapsulated in liposomes were taken up by tomato leaves, which greatly exceeded the uptake of the unencapsulated nutrients.^20^ The Fe and Mg cargo translocated to other leaves and to the plant’s roots. Similarly, in two separate studies, the foliar application of liposomes carrying iron sulfate (FeSO_4_) improved Fe delivery in basil plants, and the foliar application of selenium (Se)-loaded liposomes resulted in Se uptake into wheat plants.^19,21^ Furthermore, recent work by our group showed that encapsulating sodium bromide (NaBr) in liposomes reduced Br^–^ leaching from 90 to 45% in saturated agricultural soil and from 78 to 47% in unsaturated agricultural soil, when compared to unencapsulated NaBr.^23^ These studies demonstrate that liposomes can effectively carry agrochemical cargo, reduce cargo leaching in soil, and transport their cargo efficiently into and within plants.

In both foliar and soil applications, liposomes end up in the soil. Therefore, it is essential to investigate how their presence may alter soil biogeochemistry. Applying liposomes directly to soil could offer several potential advantages over foliar application. Unlike foliar applications, soil applications are not inherently limited by leaf surface area, where as little as 25% of the applied agrochemical reaches the plant.^24,25^ While foliar application studies are useful for assessing uptake and translocation of liposome cargo, they often overlook the fate of liposomes not adhered to or remaining on the leaf surface. Soil applications may improve delivery efficiency at the field scale, as is the case with traditional fertilizer applications. Beyond a recent study evaluating liposome transport in soil, it is unknown how the lipid component of liposomes will interact with in-situ soil microbial communities in a matrix characterized by heterogeneous soil structures, chemical compositions, and grain sizes.^23^ Studying the influence of liposomes and their cargo on soil biogeochemical cycles is a necessary step in the investigation of their efficacy as agrochemical carriers.

Because the carbon (C) and nitrogen (N) cycles are inherently linked, soil incubations are often used to assess the microbially mediated soil C and N dynamics, particularly through quantification of carbon dioxide (CO_2_) production rates alongside rates of key microbially mediated N cycling reactions including mineralization, nitrification, and denitrification (Figure 1).^26–32^ Although other studies have used soil incubations to assess the impact of various C-based agrochemical carriers (chitosan, biochar, lignite) or additives (single- and multi-walled C nanotubes, C nanoparticles, graphene nano-fertilizers) on soil microbial communities, soil respiration, and N cycling, none have evaluated liposomes.^33–36^ As lipid vesicles, liposomes contain C that may be metabolized by soil microbes and induce rapid N immobilization. This process may be important, as nitrate (NO_3_^–^) is the most mobile form of N and susceptible to leaching or denitrification losses, of which agriculture is a major contributor (Figure 1).^37^ If liposomes induce soil N immobilization, they may interrupt the rapid leaching of NO_3_^–^ and mitigate some of the associated downstream consequences including eutrophication and both groundwater and surface water contamination.^38–42^ Understanding how liposomes interact with the soil microbiome and related nutrient cycles is an important step in assessing their effectiveness as nutrient carriers.

**Figure 1:**
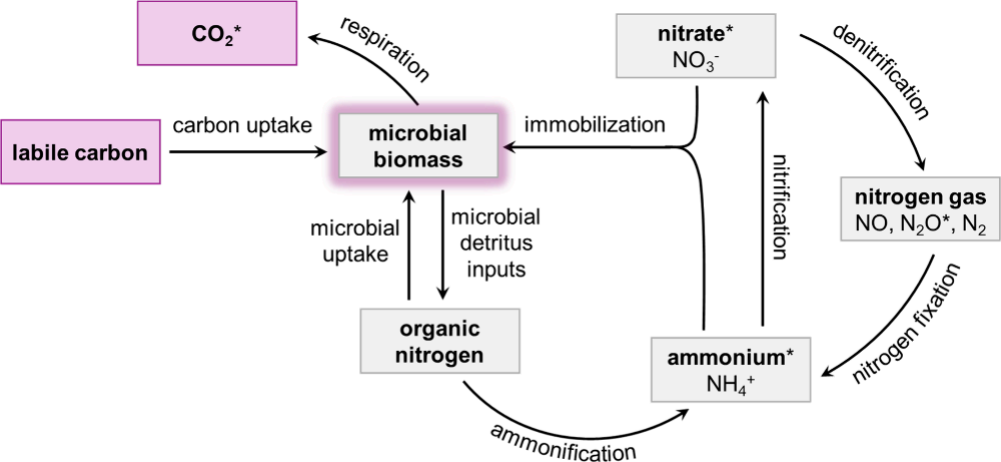
Simplified representation of the links between soil N and C cycles. Labile C provides energy to the microbial community, which increases microbial respiration and drives N cycle processes, including microbial uptake of organic N, NO_3_^–^ immobilization into microbial biomass, mineralization (ammonification and nitrification), denitrification, and N fixation. Adapted from.^43,44^ Parameters that are measured directly in this study are denoted by *.

To evaluate the interactions of liposomes with soil microbes, we conducted a 7-day soil incubation experiment using a soybean lecithin-based liposome (hereafter referred to as “liposome” or “liposomes”), loaded with and without N cargo, developed by our group. We assessed the impact of the liposomes and their cargo on soil C and N cycling through a series of measurements taken before and after the incubation period including microbial respiration (CO_2_ concentrations) and soil extractable nitrate (NO_3_^–^-N) and ammonium (NH_4_^+^-N). The relative impact on denitrification was assessed by measuring headspace N_2_O and O_2_ concentrations, as well as dual isotopes of NO_3_^–^-N extracted from soils. The study objectives were to: (1) document the extent of liposome interactions with soil microbes through the measurement of gas fluxes and (2) evaluate the impact of liposome additions on the rates of soil N cycling, including mineralization and nitrification. To accomplish these objectives, we compare two different forms of labile C additions – liposomes and glucose – and the addition of N when encapsulated in liposomes versus added outside the liposomes to understand how C lability and the encapsulation of N in liposomes contribute to the observed outcomes.

## Materials and Methods

### Soil Collection and Storage

Topsoil (0–10 cm) was collected on October 25, 2023, across three agricultural plots managed and operated within the Upper Chesapeake Bay (UCB) site of the U.S. Department of Agriculture (USDA) Long-Term Agricultural Research (LTAR) Network. Specifically, the agricultural soil was sampled from the Spring Creek watershed region of the UCB LTAR at the Kepler Research Farm, a 5-ha tract of the Pennsylvania State University’s Russell E. Larson Agricultural Research Center in Pennsylvania Furnace, PA.^45^ All plots sampled were similarly managed, having been in a continuous corn-soybean rotation prior to conversion to a no-till corn management regime in 2019. Plots are fertilized with 16.8 kg N ha^–1^, 8.96 kg P ha^–1^, and 8.96 kg K ha^–1^ each year. Much of the soil across the Spring Creek area of the UCB LTAR, including the sampled plots, is typical of the agriculturally productive karst limestone valleys of Pennsylvania, being largely comprised of the Hagerstown (Typic Hapludalf) silt loam soil series.^46^ This soil has a well-defined Ap horizon (which extends to ∼23 cm depth), is composed of 12% sand, 67% silt, and 21% clay, and has an average bulk density (ρ) of 1.56 g cm^–3^.

Briefly, soil was collected by first removing corn leaves and stubble. Shovels were used to collect the soil to a 10 cm depth. Soil was placed in 102 L plastic bins, sealed, transported to the University of Pittsburgh, and stored in a 4 °C refrigerator prior to use. Soil from across the 8 bins was mixed and homogenized by hand. Large (>1 cm diameter) rocks were removed by hand during homogenization. Average gravimetric water content (GWC) was determined by oven-drying (105 °C) 5 representative subsamples of ∼12 g soil for at least 48 h, or until constant mass was achieved.

### Liposome Production

Liposomes are composed of a lipid bilayer surrounding aqueous cargo. Soybean lecithin was used as the lipid component for liposome production. L-α-Lecithin (Product No. 429415; MilliporeSigma, St. Louis, MO, USA), a concentrate of soybean lecithin consisting of more than 94% phosphatidylcholine and less than 2% triglycerides, was used for liposome production in this study. Potassium nitrate (KNO_3_) was chosen as the N cargo for liposomes because it is easy to obtain and highly soluble. In addition, KNO_3_ supplies a second macronutrient, K, to the system, which may prove important when using liposomes as a carrier of fertilizer for plants in future studies.

Liposomes were produced using continuous flow microfluidic processing. Briefly, two parallel channels carrying aqueous cargo (here, KNO_3_) meet a third perpendicular channel carrying a stream of lipid dissolved in isopropyl alcohol (IPA) (Figure S1).^48^ At the junction, the lipid is sheathed by the streams of aqueous load and self-assembly of lipid molecules entraps the aqueous load.^49^ This method is referred to as hydrodynamic fluid focusing (HFF).^50^

Liposomes loaded with KNO_3_ (N-loaded liposomes, NL) were produced using a 3-input HFF chip (Dolomite, Royston, U.K.) (Figure S1). Aqueous KNO_3_ solution was prepared by dissolving KNO_3_ (100 mg mL^–1^) in milli-Q (MQ) water. The lipid phase was prepared by dissolving soybean lecithin (100 mg mL^–1^) in IPA. The flow rate ratio of KNO_3_:lipid was 1:1 and the total flow rate was 400 μL min^–1^. Liposomes underwent dialysis in tubing with a molecular weight cut off of 12–14 kDa (SpectraPor, Repligen, Waltham, MA, USA) in MQ water at 4 °C for 3 days to remove residual solvent or unencapsulated KNO_3_. MQ water was refreshed approximately two times per day during dialysis. Empty liposomes (EL) were prepared in the same manner as NL, but with MQ water in place of the aqueous KNO_3_ stream.

### Liposome Characterization

The C concentrations of the liposomes were determined by the non-purgeable organic carbon (NPOC) protocol on a Shimadzu TOC-L Total Organic Carbon Analyzer (Shimadzu Scientific Instruments, Columbia, MD, USA). Hydrodynamic size, polydispersity index (PDI), and zeta potential of prepared liposomes were determined by dynamic light scattering (DLS) using a Malvern Panalytical Zetasizer nano-ZS90 (Malvern Panalytical Ltd., U.K.). Zeta potential is a measurement of surface charge and can indicate likeliness for liposomes to aggregate. Liposomes were diluted 1:100 in MQ water for determination of TOC, hydrodynamic size, and PDI. Zeta potential and pH were measured on aliquots of undiluted liposomes. Nitrogen concentrations (as nitrate, NO_3_^–^-N) of the liposomes were determined using the vanadium­(III) chloride microplate method using liposomes diluted 1:50 in MQ water.^51^ Standards were prepared with the addition of empty liposomes to match the matrix for this analysis.

### Experimental Treatments

Our experimental design included 3 liposome treatment groups—N-loaded liposomes (NL), empty liposomes (EL), and N added outside empty liposomes (NEL)—and 4 control groups—a true “blank” control with MQ water only (control), an N control of KNO_3_ (N-only), a C control of glucose (G), and an N+C control composed of KNO_3_ and glucose (NG). All N was added as KNO_3_, either in solution for N-amended treatments (NEL, N-only, NG) or via liposomes, as for NL. Glucose was added as an aqueous solution. The scheme of treatments varies in contributions of N, C, or N+C as summarized in Table 1. Glucose was used as the carbon control as it is known to be a highly labile carbon source for microbes.^52^ To confirm that the liposomes were not introducing additional microbes to the incubation vials, EL and NL were also incubated alone (without soil).

**Table 1:** Soil Treatments Categorized by Their Contribution of Nitrogen (N), Carbon (C), or Both Nitrogen and Carbon (N+C)

N	C	N+C
KNO_3_ (N-only)	empty liposomes (EL)	N-loaded liposomes (NL)
	glucose (G)	N + empty liposomes (NEL)
		N + glucose (NG)

### Soil Incubation

Prepared soil (12–13 g) was incubated in the dark at 24 °C in airtight 120 mL serum bottles sealed with silicone septa for 7 days with N, C, or N+C amendments (Table 1). Carbon for C and N+C treatments was added at a rate of 800 mg C kg^–1^ dry soil as aqueous glucose (G, NG) or by addition of liposomes (EL, NL).^53^ Both the glucose (278 mM) and KNO_3_ (2.7 mM) spiking solutions were prepared in the lab using MQ water to match the measured concentrations of C and N in NL. Accordingly, N dosage was based on the N content and volume of NL used to achieve 800 mg C kg^–1^ dry soil, which was the equivalent of 2.35 mg N kg^–1^ dry soil. Five replicates were prepared for each treatment. To account for differences in soil moisture, MQ water was added to the soil to reach a target volumetric water content (VWC) of approximately 0.6 after treatment solutions were added.^54,55^

Soil NO_3_^–^-N and NH_4_^+^-N were extracted from soils using a 2 M KCl solution. A 2 M KCl solution was prepared and mixed with soil samples immediately following treatment addition (initial) and a duplicate set of treated soils following the 7-day incubation (final). Concentrations of NO_3_^–^-N and NH_4_^+^-N were measured colorimetrically on a SpectraMax iD3Multi-Mode Microplate Reader (Molecular Devices, LLC, San Jose, CA, USA) by the vanadium­(III) chloride microplate method and the salicylate microplate technique, respectively.^51,56^

### Potential Net Mineralization vs Immobilization Rates

From the measured initial and final concentrations of NO_3_^–^-N and NH_4_^+^-N in the incubation experiments, we calculated potential net N cycling rates, differentiating between potential net nitrification and ammonification rates, as equal to the difference between final and initial NO_3_^–^-N and NH_4_^+^-N concentrations (as mg N kg^–1^ dry soil day^–1^), respectively.^57^ We define mineralization as the conversion of organic N to any form of inorganic N (NO_3_^–^ and NH_4_^+^).

### Headspace Sampling

Headspace gas samples were taken from incubation vials on days 0 (background), 1, 3, and 7. After sampling the headspace, incubation vials were left open to re-equilibrate with ambient air for at least 60 min before being resealed. Gas was sampled above the vials immediately prior to sealing for the next period of incubation, on days 1 and 3, to determine if vial headspace returned to background concentrations. Gas samples were stored in evacuated vials and analyzed for CO_2_ (microbial respiration), N_2_O (possible denitrification), and O_2_ concentrations within 1 week of the day 7 sampling. Gas standards of CO_2_ (1000 ppm_v_) and N_2_O (1 ppm_v_) were injected into evacuated vials alongside actual samples during the experiment to account for any leaks during the storage period. CO_2_ and N_2_O concentrations were measured on a Shimadzu Nexis 2030 gas chromatograph (Shimazdu Scientific Instruments, Columbia, MD, USA). O_2_ concentrations were measured on a Quantek Instruments portable headspace oxygen analyzer (Model 901, Quantek Instruments, Inc., Grafton, MA, USA).

All gas concentrations were converted from ppm_v_ to μmol mL^–1^ using the ideal gas law, assuming room temperature of 25 °C and pressure of 1 atm. Concentrations for CO_2_ and N_2_O were corrected for instrument error and for vial leakiness during sample storage based on the percent difference between expected and measured values for check standards. The rate of CO_2_ production was calculated by dividing the concentration of C as CO_2_ in the vial headspace by the number of days the vial was sealed for each interval: day 0–1, day 1–3, and day 3–7. These rates are referred to as potential C mineralization rates and presented as mg C kg^–1^ dry soil day^–1^.

### Isotopic Analyses

Nitrate isotopes can be used to identify key N transformation processes, including denitrification (Figure 1). Nitrate isotopes (δ^15^N-NO_3_^–^, δ^18^O-NO_3_^–^) were measured on an Isoprime precisION isotope ratio mass spectrometer (IRMS) operating in continuous flow (CF) coupled to an iso FLOW GHG headspace analyzer (Elementar U.K. Ltd., Cheadle Hulme, Stockport, U.K.) following the denitrifier method.^58^ The denitrifier method was modified to accommodate KCl-extracted samples by preparing international reference standards in 2 M KCl solutions to matrix-match the samples and by diluting all samples and standards to the same NO_3_^–^-N concentration such that the volume of KCl injected into each vial was equal.^59,60^ International reference standards USGS-32, USGS-34, USGS-35, and IAEA-N3 were used to correct samples. Measurement accuracy was determined on repeated measures of QC standards for δ^15^N-NO_3_^–^ (0.3 ± 0.01‰) and δ^18^O-NO_3_^–^ (−1.7 ± 1.1‰) (±standard deviation, SD). Measurement precision across the study was determined on 42 repeated measures on study samples for δ^15^N-NO_3_^–^ (±0.1‰) and δ^18^O-NO_3_^–^ (±0.2‰).

### Statistical Analyses

All statistical analyses were performed using MATLAB (Version R2023b, The MathWorks Inc., Natick, MA, USA). Two sample *t* tests were used to determine statistically significant differences among initial and final NO_3_^–^-N and NH_4_^+^-N concentrations in soil and between paired treatment groups for gas concentrations. A one-way analysis of variance (ANOVA) followed by Tukey’s honest significant difference (HSD) post hoc test (*P* = 0.05) was used to compare N_2_O concentrations and potential C mineralization rates between the treatment groups. Unless otherwise noted, data is reported as the mean ± one standard error (SE).

## Results

### Liposome Physiochemical Properties

Liposome physiochemical properties are summarized in Table 2. The PDI values indicate a relatively uniform size distribution.^61^ The zeta potential indicates a negative surface charge and a propensity for aggregation over time, as it falls between −30 and +30 mV.^62^

**Table 2:** Liposome Physiochemical Properties.^a^

Property	Empty liposomes (EL)	Nitrogen-loaded liposomes (NL)
NPOC (ppm of C) (n = 5)	15,946 ± 433	12,924 ± 467
Hydrodynamic size (nm) (n = 3)	1,427 ± 140	734 ± 29
PDI (n = 3)	0.288 ± 0.064	0.354 ± 0.084
Zeta potential (mV) (n = 3)	–1.36 ± 0.2^b^	–19 ± 0.3^c^
NO_3_^–^-N (ppm) (n = 7)	0	38 ± 2
pH (n = 1)	5.4^b^	5.8^c^

a Values are reported as averages ± one SD, except for pH.

b Measured on a different batch of empty liposomes, but prepared in the same manner as used in this study.

c Measured on a different batch of N-loaded liposomes, with NO_3_^–^-N concentration of 175 ppm.

### Headspace Gas Samples

Glucose treatments had the highest average CO_2_ concentrations after the first day of incubation (22,390 ± 3262 ppm_v_ NG, 25,458 ± 2254 ppm_v_ G) (Figure S2). On day 7, liposome treatments had the highest CO_2_ concentrations, approaching or exceeding those of the glucose treatments on day 1 (22,265 ± 2257 ppm_v_ NL, 21,395 ± 2506 ppm_v_ NEL, 23,024 ± 3418 ppm_v_ EL). Concentrations of CO_2_ generally increased for liposome treatments but decreased for glucose treatments when comparing day 1, 3, and 7 samples.

Similar to CO_2_, N_2_O concentrations were highest following the first day of incubation for glucose treatments (28.4 ± 5.7 ppm_v_ NG, 34.7 ± 6.9 ppm_v_ G) (Figure 2a). Liposome treatments (NL, NEL, EL) also had the highest N_2_O concentrations on the first day, though they were less than half the concentration in glucose treatments, ranging from 5.4 to 9.3 ppm_v_. Day 3 and day 7 N_2_O concentrations did not rise above 2 ppm_v_ for any of the treatments. The average ambient concentration of N_2_O was 0.6 ppm_v_. Oxygen concentrations were below ambient levels (205,800 ppm_v_) for all samples, ranging from 169,250 to 195,200 ppm_v_ (Figure 2b).

**Figure 2:**
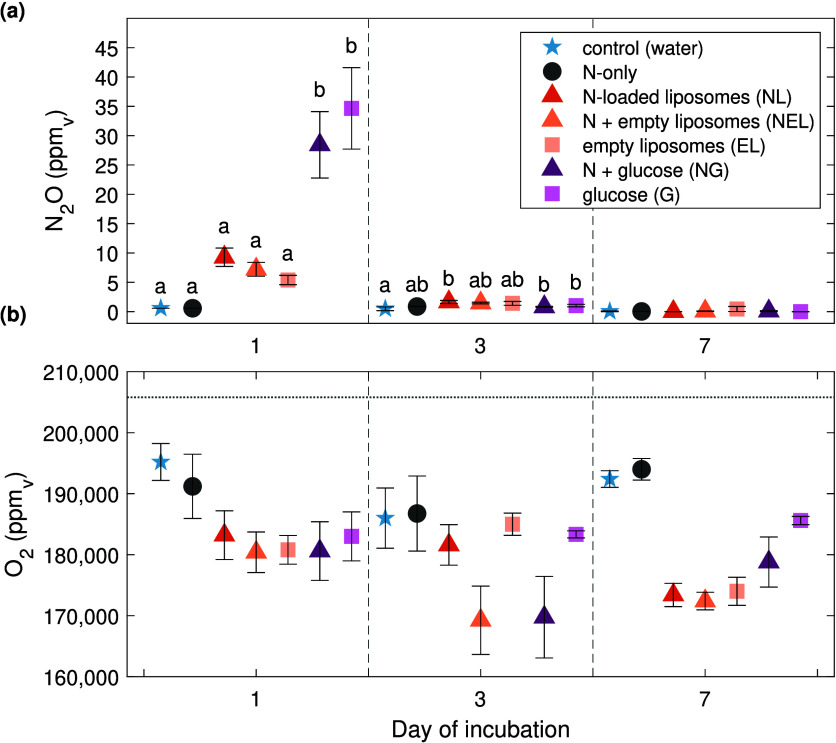
Concentrations of (a) N_2_O and (b) O_2_ expressed as partial pressures (ppm_v_) measured in incubation vial headspace samples collected on days 1, 3, and 7. Treatments are grouped by symbols: control (star), N (circle), C (square), N+C (triangle) (*n* = 5 for each treatment, except day 3 O_2_: G *n* = 3; N-only, NG, and NEL *n* = 4)). The horizontal dashed line on plot (b) represents the average ambient O_2_ concentration measured (205,800 ppm_v_). Error bars represent ± one SE. Letters indicate statistically different means between treatments on the same day for figure (a) (*P* < 0.05).

### Potential C Mineralization Rates and C Mass Balance

Potential C mineralization rates are expressed as CO_2_ production rates for each interval during which incubation vials were sealed (Figure 3a). While potential rates of C mineralization are usually expressed as averages over the entire incubation period, looking at the rates for the three time intervals here allows us to assess differences in C substrate lability (glucose vs liposomes) with finer temporal resolution.^32^ All glucose and liposome treatments had potential C mineralization rates that were significantly higher than the control on all days (*P* < 0.05), except for glucose on day 7 (*P* > 0.05). Among the two C-only treatments, glucose (G) had a significantly higher potential C mineralization rate on day 1 than empty liposomes (EL) (*P* > 0.05). The encapsulation of N in liposomes (NL) did not result in significantly different potential C mineralization rates when compared to the addition of N outside of empty liposomes (NEL) on any day (*P* > 0.05). When averaged across the 3 liposome treatments (NL, NEL, EL) and 2 glucose treatments (NG, G), there was no statistically significant difference in potential C mineralization rates or average total mass of C respired (Figure 3b) exceeding the control (*P* > 0.05). The respired C amounted to an average of 29% and 34% of the C added (8 mg) for liposome and glucose treatments, respectively.

**Figure 3:**
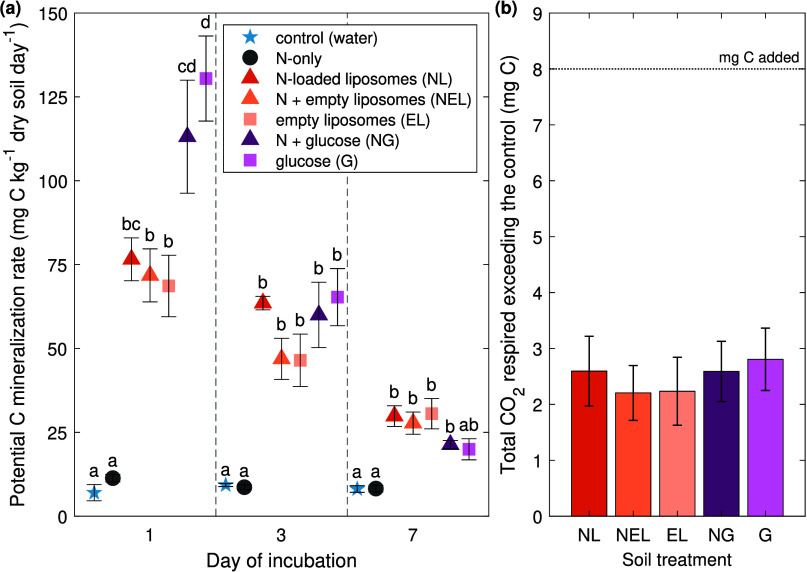
(a) Average potential carbon mineralization rates across the 7-day incubation. (b) Average total C respired (mg) above the control for treatments which received C or N + C. The horizontal dashed line indicates the 8 mg of C added for each treatment. Treatments are grouped by symbols (control (star), N (circle), C (square), N + C (triangle)) and colors in order from left to right: control (water), N-only, NL (N-loaded liposomes), NEL (N + empty liposomes), EL (empty liposomes), NG (N + glucose), and G (glucose) (*n* = 5 for each treatment). Error bars represent ± one SE. Letters on (a) indicate statistically different means between treatments on the same day (*P* < 0.05).

### Soil Extractable Inorganic N

Soil extractable nitrate (NO_3_^–^-N) concentrations varied markedly between pre- and post-incubation. Nitrate concentrations were significantly lower on day 7 (final) compared to day 0 (initial) for all treatments containing either liposomes or glucose (NL 86%, NEL 71%, EL 88%, NG 75%, and G 87% reduction from intital to final, respectively) (*P* < 0.0001 NEL; *P* < 0.00001, NL, EL, NG, G) (Figure 4a). Soil extractable NO_3_^–^-N increased by 13% in the control and 16% in the N-only treatment. The magnitude of change in soil extractable NH_4_^+^-N was nearly 4 times lower than that of soil extractable NO_3_^–^-N. Significant decreases in NH_4_^+^-N of 21, 21, and 18%, respectively, were observed for N-only, NL, and NEL treatments (*P* < 0.05, *P* < 0.05, and *P* < 0.001, respectively) (Figure 4b). In contrast, soil extractable NH_4_^+^-N increased in the other treatments (control, EL, NG, G), although the increase was not statistically significant.

**Figure 4:**
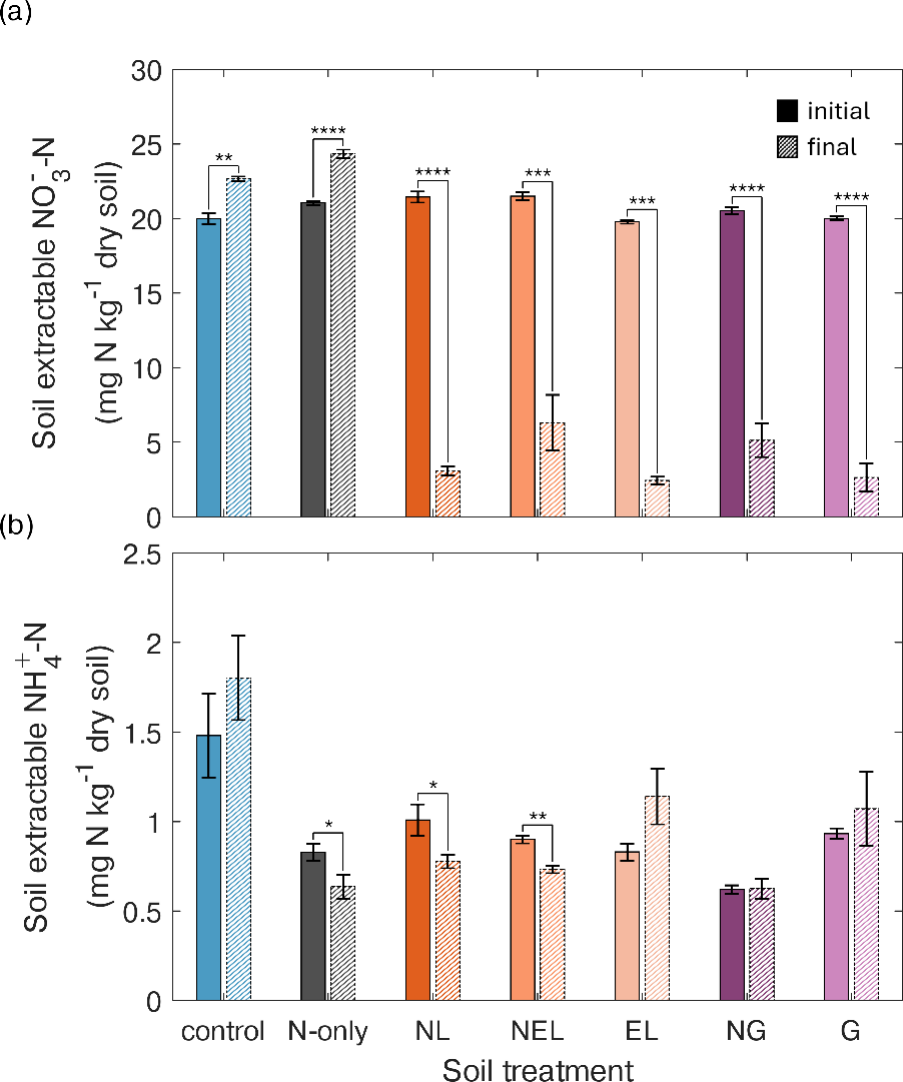
Soil extractable (a) nitrate and (b) ammonium before (initial) and after (final) a 7-day incubation. Note the different scale for the *y*-axes in (a,b). Treatments from left to right: control (water), N-only, NL (N-loaded liposomes), NEL (N + empty liposomes), EL (empty liposomes), NG (N + glucose), and G (glucose) (*n* = 5 for each treatment). Error bars represent ± one SE. Asterisks indicate statistically different means between initial and final for each treatment (*P < 0.05, **P < 0.001, ***P < 0.0001, ****P < 0.00001).

### Potential Net N Mineralization vs Net Immobilization Rates

Potential net N mineralization and immobilization rates were calculated based on the changes in soil inorganic N occurring between day 0 and day 7, where a net increase (positive; >0) in soil inorganic N signifies net mineralization and a net decrease (negative; < 0) signifies immobilization. Table 3 presents these potential rates. All C (EL, G) and N+C (NL, NEL, NG) treatments had potential net N immobilization rates ∼ 5.5 times higher than potential net mineralization rates of the control (water) and N-only treatment (KNO_3_). There was no significant difference between any liposome or glucose treatment (*P* > 0.05).

**Table 3:** Average Potential Net N Mineralization and Immobilization Rates across the 7-day Incubation Period (±One SE)^a^

Treatment	Potential net N Mineralization (>0) or Immobilization (<0) Rates (mg N kg^–1^ dry soil day^–1^)
control (water)	0.42 ± 0.08
N-only	0.44 ± 0.06
N-loaded liposomes (NL)	–2.6 ± 0.06
N + empty liposomes (NEL)	–2.2 ± 0.3
Empty liposomes (EL)	–2.4 ± 0.04
N + glucose (NG)	–2.2 ± 0.2
Glucose (G)	–2.5 ± 0.1

a *n* = 5 for each treatment.

## Discussion

Liposomes are promising tools to carry agrochemicals to soil and plants, yet their impacts on soil biogeochemical cycles are unknown. We sought to determine if and how the addition of C from the soybean lecithin liposomes altered microbially driven C and N cycling in an agricultural soil. The results of this study demonstrate that: (1) microbes consume liposomes as a C source, similar to glucose, but the initial rate of consumption differed significantly between these C substrates; (2) the addition of liposomes to soil significantly alters rates of N mineralization and immobilization; and (3) the addition of N to soil inside (NL) or outside (NEL) of the liposome does not result in significant differences in potential net C or N cycling rates.

### Liposomes are Metabolized by Soil Microbes

The results of our soil incubation experiments suggest that the liposomes, produced with soybean lecithin and rich in phospholipids, were consumed by soil microbes. Elevated CO_2_ concentrations in the C (EL, G) and N + C (NL, NEL, NG) amended groups in comparison to the control (water) treatment indicate microbial metabolism of the provided C (liposomes or glucose) (Figure S2). Increased CO_2_ production was not driven by N addition alone, as demonstrated by the significantly higher potential C mineralization rates for all C (EL, G) and N + C (NL, NEL, NG) treatments compared to the N-only treatment (except G, day 7) (*P* < 0.05), which was not statistically different than the water control on any day (*P* > 0.05) (Figure 3a).

The rate of CO_2_ production, calculated as a potential C mineralization rate, was fastest for liposome (NL, NEL, EL) and glucose (NG, G) treatments during the first day of incubation (Figure 3a). After 1 day of incubation, glucose treatments (NG, G) had significantly higher potential C mineralization rates than NEL and EL treatments (*P* < 0.05), though the rates of NL and NG were not significantly different from each other (*P* > 0.05). The faster CO_2_ production rate for glucose treatments (NG, G) compared to their equivalent liposome treatments (NEL, EL, respectively) after the first day indicates that glucose is more favored by soil microbes (i.e., more labile) than liposomes. Given that glucose is a simple sugar and a primary source of energy for microbes, this observed rapid consumption was expected.^52^ Between days 1 and 3, the potential C mineralization rate slowed for all liposome (NL, NEL, EL) and glucose (NG, G) treatments, such that they were not significantly different between the two C substrates (*P* > 0.05). The same was true for days 3–7, though the glucose treatment (G) was no longer significantly greater than the control or N-only treatments (*P* > 0.05). Averaging the 7 days, we observed no significant difference in average potential C mineralization rates or total mass of C respired (exceeding the control) when comparing liposomes and glucose (Figure 3b). Other studies comparing glucose to acetate, cellulose, lignin, or other C substrates also show that the increase in CO_2_ production varies based on C substrate type.^30,63,64^ Finer resolution data (minutes to hours) is needed to better understand this rapid metabolism of phospholipids supplied via liposomes. Moreover, of the total C applied, only 29% of liposome C and 34% of glucose C was respired, indicating that much of the substrate may remain in the soil past 7 days. Further work is needed to understand the accessibility and metabolism of lingering liposome-supplied C beyond a 7-day incubation period and the mechanisms driving short-term and long-term release or retention of liposome cargo in soil systems.

### Liposome Additions Increase N Cycling Rates and Induce Net N Immobilization

The addition of liposomes to soil resulted in increased potential N transformation rates. We used the potential net mineralization or potential net immobilization rates to approximate the relative contributions of N transformation processes across the different treatments. The addition of C or N + C led to net N immobilization, indicating that soil inorganic N was immobilized in microbial biomass (Table 3). No statistically significant differences in potential net N immobilization rates were observed when comparing the different C substrates (liposomes vs glucose) (*P* > 0.05). In contrast, the addition of water (control) or KNO_3_ (N-only) led to net N mineralization at a rate 5–6 times slower than the potential net N immobilization rates of the C and N + C groups. From the control and N-only treatment, we learned that in this soil, in the absence of new C additions, microbes are more likely to mineralize organic N and increase the NH_4_^+^ and NO_3_^–^ pools available for plant uptake or leaching.

The net decline in extractable inorganic N in soils treated with either C or N + C could reflect several processes including immobilization, denitrification, and nitrification. The net decrease of soil inorganic N, mainly NO_3_^–^-N (Figure 4a), was driven by the addition of C to the soil, as revealed by the opposite trend in the control and N-only treatment. Our data suggests that the supply of C (liposomes or glucose) was readily metabolized by soil microbes, which led to net N immobilization via N immobilization into microbial biomass or denitrification (Figure 1). Many studies have similar results, demonstrating that the addition of labile C, such as glucose, sucrose, or acetate, to soil results in quick (0–3 days) microbial immobilization of soil NO_3_^–^-N.^26–29,26–29,65–67^ Additionally, phospholipid chains, though a less labile form of C than glucose, are readily broken down within hours to days, as evidenced by the rapid oxidation of phospholipids in soil microbial necromass.^68–70^ Recous et al. primarily attributed decreases in NO_3_^–^-N to microbial immobilization, although some decreases occurred due to denitrification and volatilization.^27^

A portion of the net decrease of soil inorganic N in our study was due to gaseous loss as N_2_O, which is known to be a byproduct of both denitrification and nitrification of soil N.^71,72^ The N_2_O produced (Figure 2a) accounted for an average of 0.5 and 2.5% of the net decrease of soil inorganic N for liposome and glucose treatments, respectively. Oxygen concentrations measured here (16.9–19.5%, Figure 2b) were unlikely to be low enough to support denitrification, which requires low oxygen or anoxic conditions. To further investigate whether denitrification was a significant source of inorganic N decreases in the amended soils, we measured the δ^15^N and δ^18^O of NO_3_^–^ in initial and final KCl extracts. The isotopic data did not exhibit the typical denitrification signal of enrichment in either δ^15^N or δ^18^O (Figure S3). Rather, we observed a decrease in δ^15^N for all liposome treatments (NL, EL, NEL) from initial to final, and little change in δ^15^N and δ^18^O for all other treatments. Although denitrification may have occurred in low oxygen microsites within the soil, it is more likely that nitrification produced the N_2_O observed here based on sufficient O_2_ availability and homogenization of soil prior to use.^72,73^ Other processes that could have contributed to the observed increase in N_2_O concentrations include dissimilatory nitrate reduction to ammonium, nitrifier denitrification, and abiotic decomposition of ammonium nitrate.^71–74^ These processes may have played a role in the observed changes in δ^15^N and δ^18^O of NO_3_^–^. Finally, N can also be immobilized through abiotic reactions within seconds, though we would expect to see more NO_3_^–^-N immobilization in both the control and N-only treatment if that process was a substantial contributor here.^75^ We conclude that immobilization was likely the dominant process driving soil NO_3_^–^-N declines during the incubations.

### The presence of N Inside or Outside Liposomes Did Not Affect C and N Cycling Outcomes

Finally, whether NO_3_^–^-N was present inside or outside liposomes appears not to alter the microbial response of C or N cycling. We found no statistically significant differences in C mineralization rates (Figure 3a), N_2_O production (Figure 2a), or net soil N immobilization rates (Table 3) between the treatments when liposomes were added alone (EL), with N inside (NL), or with N outside the liposome (NEL) (*P* > 0.05). These results suggest that the encapsulation of N in liposomes does not influence how either C or N is processed by microbes. These similarities may be due to the relatively low N addition (2.35 mg N kg^–1^ dry soil) compared to the C addition (800 mg C kg^–1^ dry soil), a C:N ratio of ∼340. Varying the concentration of the N load in the liposome would alter the C:N ratio of the amendment and could produce different outcomes. Amending agricultural soils with organic matter (C inputs) is a well-studied approach to reduce N leaching from fields. Both the C:N ratio and the form of C drive N immobilization, with C:N higher than 20–40 and more labile C amendments (i.e., low lignin content) inducing higher rates of N immobilization.^66,76–79^ Further work is needed to understand if tuning liposome C:N ratios by encapsulating higher or lower concentration N could be used to target specific outcomes for soil N.

### Implications for Use of Liposomes in Agriculture

Utilizing liposomes as carriers for N offers a potential strategy to decrease N losses in agricultural soils by facilitating the immobilization of both supplied and existing soil NO_3_^–^. Notably, the observed decreases in soil inorganic N in our experiment were greater than the 2.35 mg N kg^–1^ dry soil added in N and N+C treatments, indicating that liposome additions immobilize the existing pools of soil inorganic N, not only the N that they supply. The strength of this immobilization is evident by the observed 71–88% reduction of available soil NO_3_^–^-N following liposome application to the soil, as well as the δ^15^N-NO_3_^–^ and δ^18^O-NO_3_^–^ values and the fractional (0.5%) contribution of N_2_O to the net decrease in soil inorganic N which suggest widespread denitrification did not occur. Our results align with other studies that have found that amending soil with C inputs, including glucose, triggered rapid microbial immobilization of NO_3_^–^-N, underscoring the capacity of liposome C to modify soil N dynamics in a manner comparable to other known labile C sources.^26,27,29,63^ The turnover of microbial biomass, typically within days to one year for agricultural soils, will determine how quickly this temporarily immobilized N is made bioavailable to plants again as NH_4_^+^-N; further work is needed to understand the microbial turnover rate under these experimental conditions.^26,64,80–90^ By acting as an available C source, liposomes drove increased microbial immobilization of soil inorganic N which may result in greater NO_3_^–^ retention and reduce leaching losses in an agricultural setting. Building on this outcome, the application of liposomes to soil may be timed in such a way to take advantage of this rapid N immobilization in agricultural fields.

To fully realize the potential effects of liposome-based N delivery, further work is needed to understand how soil microbes and plants may compete for liposome supplied N. Nitrogen availability is critical for optimal crop growth and yield, and understanding how rates of liposome induced microbial N immobilization compare to plant uptake of liposomes, as well as how quickly this immobilized N becomes remineralized and bioavailable to plants, is essential to understanding the outcome of using liposomes to deliver plant nutrients in the soil. Future work should focus on quantifying biomass turnover rates in liposome treated soils, tracing liposome supplied N in plant–soil systems, and investigating the availability of phosphorus from liposome lipids.

Together, our results suggest liposomes may serve as a tool for strategically manipulating microbial nutrient cycling and increasing N retention in agricultural soils. Furthermore, this study highlights the importance of considering how liposomes and other emerging carriers of agrochemicals, particularly those that are made of C, alter soil biogeochemical cycles.

## Supplementary Material



## Data Availability

The data associate with this study are available at the following doi: 10.6084/m9.figshare.30667682.

## Notes

Presentation of the hydrodynamic fluid focusing microchip used for liposome production, concentrations of CO_2_ in incubation vial headspace samples each day, and isotopic data for nitrate in KCl extracts (PDF)

## ●

Stanford Doerr School of Sustainability, Stanford University, Stanford, CA 94305, United States

## a

Center for Ecosystem Science and Society, Northern Arizona University, Flagstaff, AZ 86001, USA.
