# Supplementary material for: A Lecithin Liposome Stimulates Soil Microbial Respiration and Nitrate Immobilization
Source: ACS Agric Sci Technol. 2025 Nov 24;5(12):2509–18. doi: 10.1021/acsagscitech.5c00587 (PMC12709579; doi:10.1021/acsagscitech.5c00587)
Supplement: Supplementary file 1 [file as5c00587_si_001.pdf]

Supporting information for:

A lecithin liposome stimulates soil microbial respiration and nitrate immobilization

Camille R. Butkus[1#*], Julie N. Weitzman[1†], Alireza Mohammadzadeh[2], Patrick J. Dunn[3], Jason P. Kaye[4], Leanne M. Gilbertson[3,5], Steven R. Little[2,6-11], Emily M. Elliott[1,3]

[1]Department of Geology and Environmental Science, University of Pittsburgh, Pittsburgh, PA 15260, USA

[2]Department of Bioengineering, University of Pittsburgh, Pittsburgh, PA 15260, USA

[3]Department of Civil and Environmental Engineering, University of Pittsburgh, Pittsburgh, PA 15260, USA

[4]Department of Ecosystem Science and Management, The Pennsylvania State University, University Park, PA 16802, USA

[5]Department of Civil and Environmental Engineering, Duke University, Durham, NC 27708, USA

[6]Department of Department of Chemical and Petroleum Engineering, University of Pittsburgh, Pittsburgh, PA 15260, USA

[7]Department of Clinical and Translational Science, University of Pittsburgh, Pittsburgh, PA 15260, USA

[8]McGowan Institute for Regenerative Medicine, University of Pittsburgh, Pittsburgh, PA 15260, USA

[9]Department of Immunology, University of Pittsburgh, Pittsburgh, PA 15260, USA

[10]Department of Pharmaceutical Sciences, University of Pittsburgh, Pittsburgh, PA 15260, USA

[11]Department of Ophthalmology, University of Pittsburgh, Pittsburgh, PA 15260, USA

[#]Present address: Center for Ecosystem Science and Society, Northern Arizona University, Flagstaff, AZ 86001, USA

[†]Present address: Stanford Doerr School of Sustainability, Stanford University, Stanford, CA 94305, USA

*Corresponding author: crb181@pitt.edu

This supplementary file contains:

Figure S1: Hydrodynamic fluid focusing microchip used for liposome production.

Figure S2: Concentrations of CO_2 expressed in incubation vial headspace samples collected on days 1, 3, and 7.

Figure S3: Isotopic data for nitrate in KCl extracts.

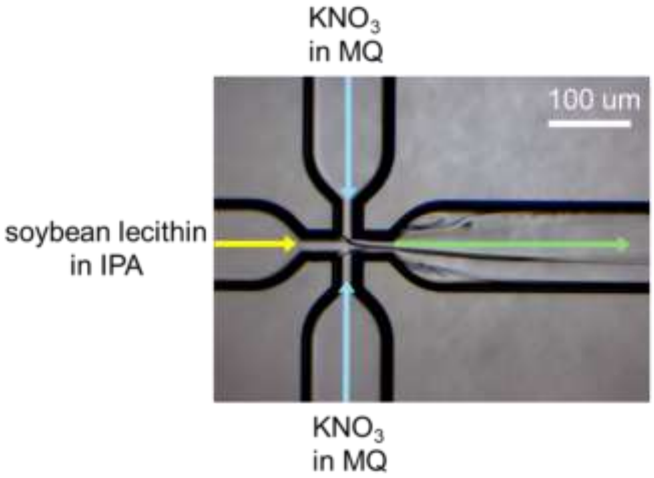

Figure S1. Image of a hydrodynamic fluid focusing (HFF) microchip used to produce liposomes. Arrows indicate the direction of flow, where the lipid (soybean lecithin) in isopropyl alcohol (IPA) (yellow arrow) meets a 2 perpendicular streams of aqueous cargo (here, KNO_3 for N-loaded liposomes; blue arrows) and self-assembles into liposomes loaded with cargo (green arrow).

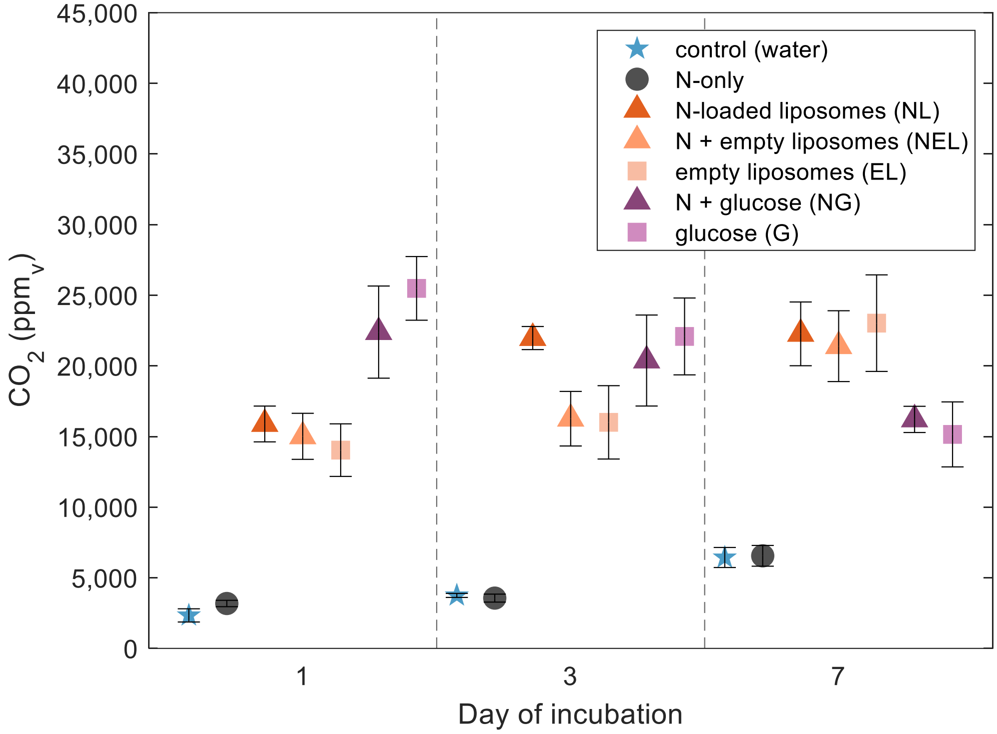

Figure S2. Concentrations of CO_2 expressed as partial pressures (ppm_v) measured in incubation vial headspace samples collected on days 1, 3, and 7. Treatments are grouped by symbol (control (star), N (circle), C (square), N+C (triangle)) and color in order from left to right: control (water), N-only, NL (N-loaded liposomes), NEL (N + empty liposomes), EL (empty liposomes), NG (N + glucose), and G (glucose) (n=5 for each treatment). Error bars represent ± one standard error

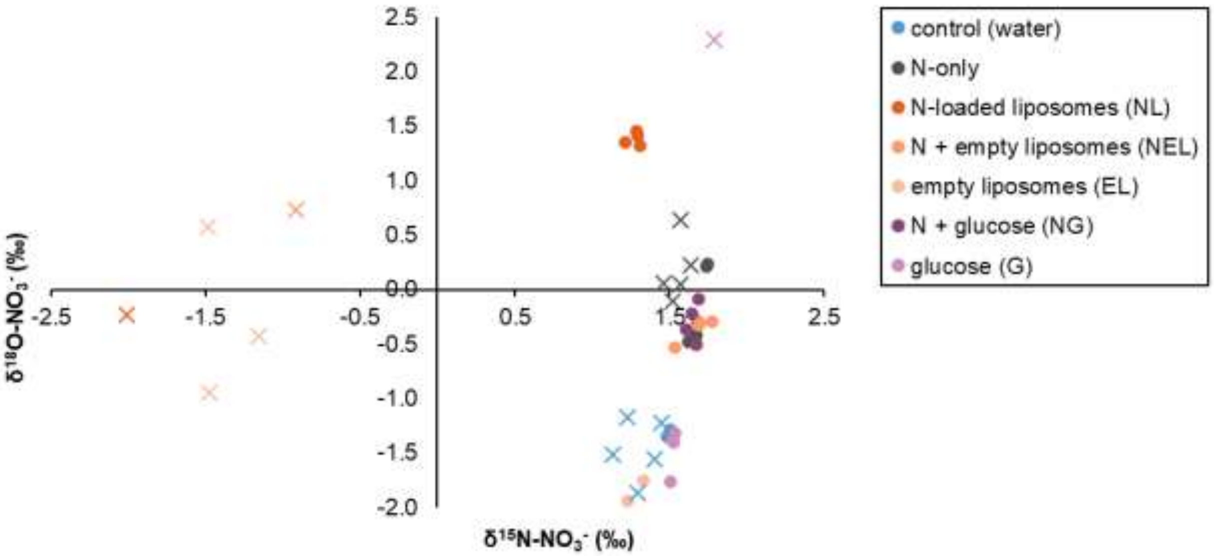

Figure S3. Dual isotopes of NO_3^- (i.e., $\delta^{15}N–NO_3^-$ vs. $\delta^{18}O–NO_3^-$) in 2 M KCl extracts collected at the start (circle) and end (x) of the soil incubation experiments for each treatment group.
